# Infected Aortic Aneurysm Caused by *Streptococcus zooepidemicus*: A Case Report and Literature Review

**DOI:** 10.3400/avd.cr.20-00133

**Published:** 2021-03-25

**Authors:** Yuji Matsubayashi, Noriyuki Takashima, Yasuo Kondo, Hodaka Wakisaka, Tomoaki Suzuki

**Affiliations:** 1 Division of Cardiovascular Surgery, Department of Surgery, Shiga University of Medical Science, Otsu, Shiga, Japan

**Keywords:** *Streptococcus zooepidemicus*, infected aortic aneurysm, higher mortality

## Abstract

A 66-year-old Japanese male working at a stable developed abdominal pain and fever and was brought to the emergency department. The computed tomography scan revealed an aneurysm of approximately 70 mm in diameter, with an irregular border, at the infrarenal abdominal aorta. Emergency surgery was performed with a bifurcated Dacron graft. *Streptococcus zooepidemicus* was observed on the aneurysm wall. He was discharged in good condition and was prescribed oral amoxicillin/clavulanic acid for 4 months. He has remained well and did not develop inflammation. Evaluation of patient history and data, including the consumption of unpasteurized dairy food, occupation, and direct contact with animals, is important for an early diagnosis, a prompt surgery, and an appropriate antibiotic therapy.

## Introduction

*Streptococcus zooepidemicus* is a Lancefield group C β-hemolytic streptococcus.^1)^ It is well known to cause respiratory tract and wound infections in young horses.^1)^ Although it rarely infects humans, it may opportunistically infect humans with underlying diseases.^1)^ It is mainly transmitted through close contact with animals and consumption of unpasteurized dairy products.^2)^ The possible effects of *S. zooepidemicus* infection in humans include nephritis, arthritis, meningitis, pneumonia, infected aortic aneurysm, infective endocarditis, and sepsis.^1)^ When an infected aortic aneurysm (AA) is caused by *S. zooepidemicus*, a poor prognosis is expected.^3)^ Its clinical course, prognosis, and standard treatment are still largely unknown due to only a few cases being reported in the literature. To understand the traits of infected AA caused by *S. zooepidemicus*, we reviewed the literature and collected 12 cases. Those cases confirm the value of taking a full history for early diagnosis, prompt surgery, and accurate antibiotic treatment.

## Case Report

A 66-year-old Japanese man working at a stable was brought to the emergency department of a peripheral hospital due to complaints of abdominal pain, fever, and weight loss. He had already been followed by the same hospital for 2 months due to rib fracture caused by a fall. His past medical history was not significant in other respects. The computed tomography (CT) scan revealed an infrarenal abdominal AA of about 70-mm diameter. He was transferred to our department with a saline drip.

**Figure 1:**
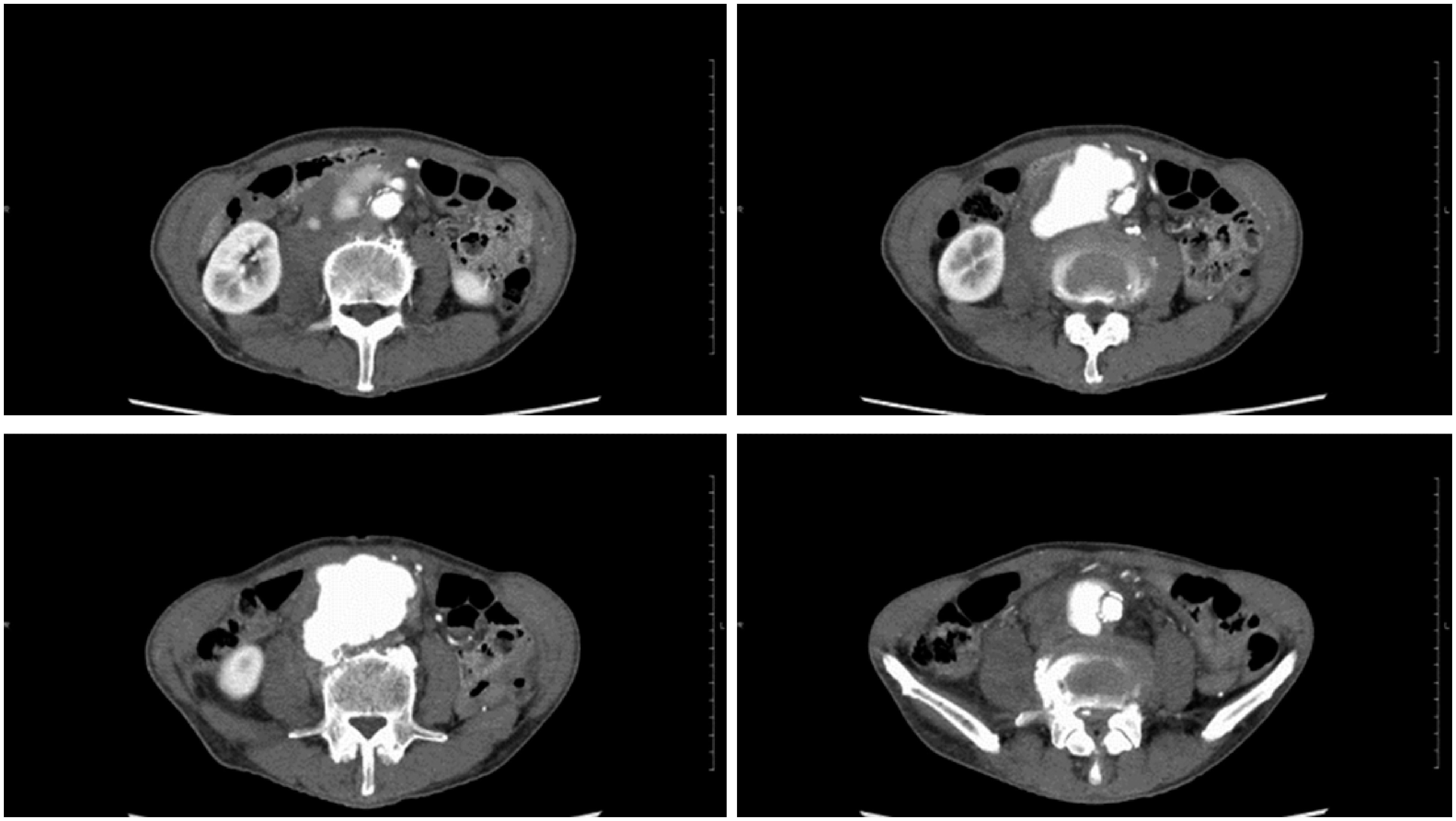
Fig. 1 A contrast-enhanced computed tomography scan revealed an abdominal aortic aneurysm with a saccular, multilocular appearance and an irregular border. The aneurysm extended from the infrarenal abdominal aorta to just above the bifurcation. Rupture of the aneurysm was suspected.

He was hemodynamically stable upon his arrival at our hospital, and his blood pressure was 112/73 mmHg; heart rate, 84 bpm; body temperature, 38.1°C; and SO_2_, 99% (room air). The blood test revealed mild anemia (Hb 9.8 g/dL), normal white blood cell count (7400/µL), and increased C-reactive protein (CRP) level (11.08 mg/dL). Physical examination revealed emaciation and a pulsatile mass in his lower abdomen. The contrast-enhanced CT scan revealed an aneurysm with an irregular border (**Fig. 1**).

**Figure 2:**
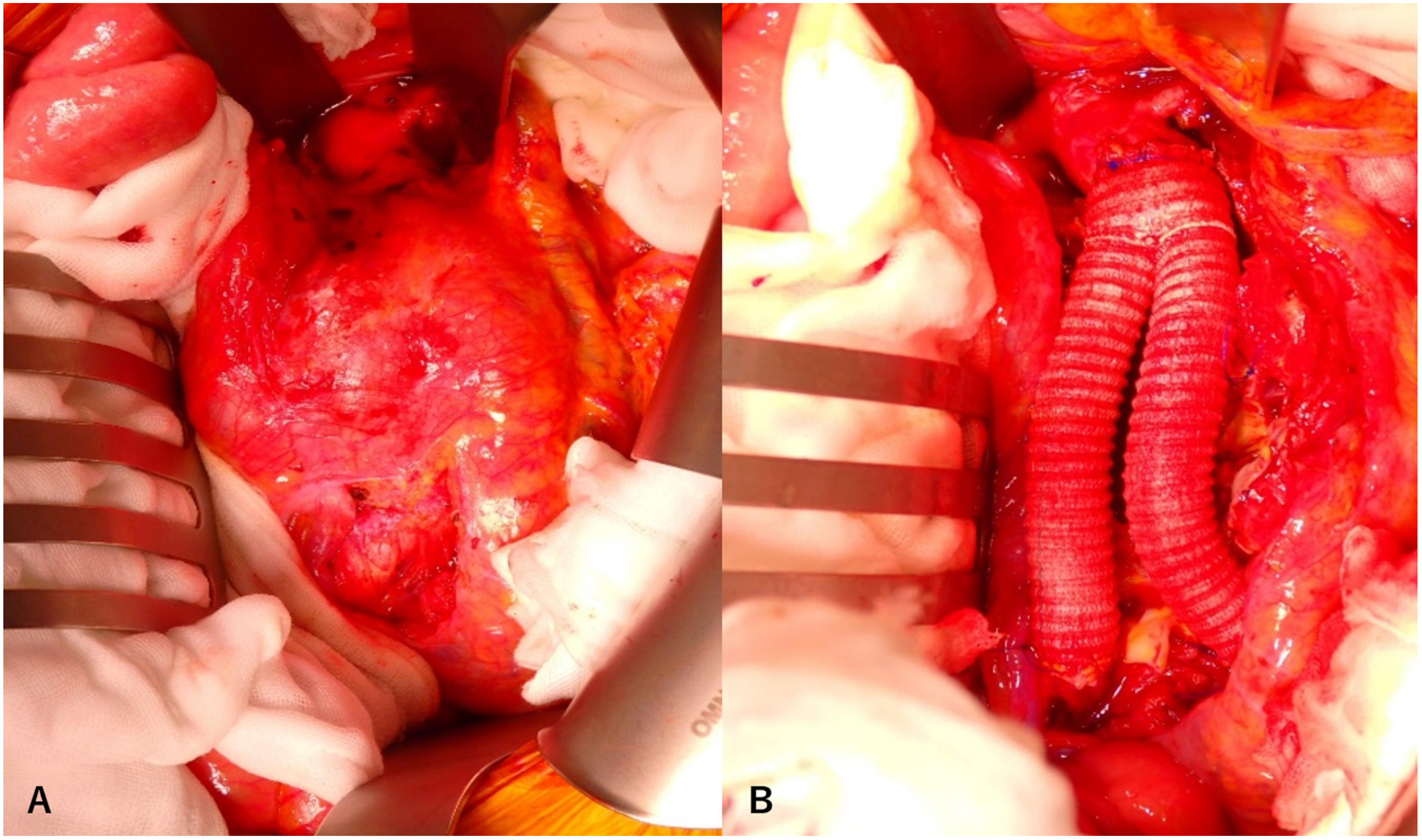
Fig. 2 Emergency surgery was performed. The aneurysm strictly adhered to the duodenum, inferior vena cava, and retroperitoneum. (**A**) The aneurysm strictly adhered to the duodenum, inferior vena cava, and retroperitoneum. (**B**) We carefully resected the aneurysm and replaced it with a bifurcated Dacron graft.

Emergency surgery was performed through midline laparotomy, which exposed an aneurysm strictly adhering to the duodenum, inferior vena cava, and retroperitoneum (**Fig. 2A**). The aneurysm was cautiously resected and replaced with a bifurcated Dacron graft (J-graft 18–11 mm, Japan Lifeline Co., Ltd., Tokyo, Japan) (**Fig. 2B**). The normal intimal wall existed only at the posterior aortic wall, which indicated pseudoaneurysm. A wide-ranging debridement of the infected tissue, including the aneurysm wall, was performed with copious irrigation. The operation time was 143 min. The amount of bleeding was 1296 mL. Four red blood cell units (560 mL) were transfused.

*S. zooepidemicus* was identified from the aneurysm wall extracted during the operation, which led to the definitive diagnosis of an infected abdominal AA. However, blood culture was negative. The strain proved vulnerable to all the major antibiotics in the antibiotic susceptibility testing. Intravenous administration of cefazolin was empirically started before the operation and continued for 3 weeks. The patient’s body temperature fell into the normal range on post-operative day (POD) 8. His CRP level also became normal from POD 20. Intravenous cefazolin was replaced with oral amoxicillin/clavulanic acid and continued for 4 months until the patient chose to discontinue this prophylaxis. The CT scan revealed no inflammation tissue around the residual aortic wall, duodenum, and retroperitoneum. He was discharged in good condition on POD 23. No recurrence of inflammation was observed in the 6 months following the operation.

## Discussion

*S. zooepidemicus* infection is common in animals, such as horses, cattle, sheep, and pigs, but rare in humans.^1)^ When humans are infected, they can develop diseases, such as bacteremia, endocarditis, and infected AA.^1)^

Several case reports on bacteremia or *S. zooepidemicus* outbreaks have been observed. However, there have only been a few cases associated with *S. zooepidemicus* infection leading to infected AA. Although it seems potentially lethal, little is known about its clinical course, prognosis, and optimal treatment. For further information, we searched Google Scholar for case reports using the terms *S. zooepidemicus* and aneurysm between 1980 and 2020. This yielded 162 papers, among which were reports of 11 cases of infected AA caused by *S. zooepidemicus*, with information on underlying diseases, operations, and outcomes, as presented in the **Table 1**.^2–8)^

**Table 1:** Table 1 A summary of 12 cases diagnosed with infected aortic aneurysm induced by *S. zooepidemicus*

Case	Year	Reference	Age/Sex	Underlying disease	Source of isolate	Cause of infection	Diagnosis	Operation	Antibiotics	Outcome
1	1982	5)	65/M	none	aortic wall	unknown	infrarenal aneurysm	bifurcated Dacron graft	cephalothin, gentamicin, metronidazole followed by iv. penicillin for 4 weeks	well
2	1982	5)	62/M	cirrosis of the liver portal hypertension	aortic wall	unknown	infrarenal aneurysm	bifurcated Dacron graft	iv. amoxycillin and gentamicin	died due to cardiac failure on POD 10
3	1988	6)	73/M	ischaemic HD	vegetations blood	unpasteurized milk	AAA meningitis endocarditis	none	unknown	died due to raptured AAA
4	1990	7)	62/M	cirrosis of the liver portal hypertension	aortic wall blood	undercooked pork	AAA	bifurcated Dacron graft	iv. amoxycillin and gentamicin	died
5	1990	7)	65/M	intravascular infection	aortic wall blood	undercooked pork	AAA	bifurcated Dacron graft	iv. benzylpenicillin	well
6	2006	3)	59/M	HT, DM	blood	fresh cheese	infrarenal aneurysm	aortobifemoral graft	iv. penicillin and gentamicin for three weeks oral TMP-SMX for 6 months	well
7	2006	3)	68/M	HT, DM, COPD	blood	fresh cheese	suprarenal aneurysm	aortoaortic graft left renal artery reimplanted	iv. penicillin and gentamicin	died due to small intestinal necrosis on POD 3
8	2006	3)	69/M	HT, DM	blood	fresh cheese	descending TAA	thoracic endograft	iv. penicillin and gentamicin	died due to sepsis on POD 2
9	2013	8)	49/M	none	aortic wall	horse trainer trauma	AAA	Y-prosthesis	iv. piperacillin-tazobactam followed by iv. penicillin later	well
10	2019	2)	70/M	hypothyroidism, DLP	blood	taxidermist contact with a deceased horse	AAA right CIAA	autologus vein reconstruction (both superficial femral veins)	iv. penicillin for 6 weeks oral penicillin at home	well
11	2020	4)	79/M	DM, DLP, MG	blood	unknown possibly spinal facet injections	AAA	EVAR	iv. clindamycin for 6 weeks oral clindamycin for 3 months	well
12	2020	present case	66/M	none	aortic wall	horse keeper trauma	infrarenal aneurysm	bifurcated Dacron graft	iv. cefazolin for 2 weeks oral clavulanate/amoxicillin for 4 months	well

HT: hypertension; DM: diabetes mellitus; COPD: chronic obstructive pulmonary disease; DLP: dyslipidemia; HD: heart disease; TAA: thoracic aortic aneurysm; AAA: abdominal aortic aneurysm; TMP-SMX: Trimethoprim-sulfamethoxazole; CIAA: common iliac artery aneurysm; MG: myasthenia gravis; EVAR: endovascular aneurysm repair; POD: post-operative day

All patients were male, with a mean age of 66 (SD: ±7.2) years. The overall mortality was 42% (5 out of 12 patients). Among those 12 patients, only 3 (25%) had no underlying disease, and none of those 3 died. Among the other 9 patients (75%) who had underlying diseases, 5 (56%) died.

The most common organism associated with infected AA is *Staphylococcus aureus*, which accounts for some 30% of cases, followed by *Salmonella spp*.^9)^ Infected AA is associated with high mortality rates: around 21%–30% with medical treatment, reported by Kim,^9)^ and 13% with surgical treatment, reported by Yeager et al.^10)^ The overall mortality of infected AA caused by *S. zooepidemicus* is higher than that of infected AA caused by *Staphylococcus aureus* and *Salmonella spp.*

Patients infected with *S. zooepidemicus* are likely to have underlying medical conditions.^1)^ *S. zooepidemicus* is generally regarded as an opportunistic pathogen.^4)^ In this review, we found that patients with underlying diseases are more vulnerable to *S. zooepidemicus*, and so to infected AA.

Our patient had been working as a horse trainer and had no medical conditions. This is a remarkable case as *S. zooepidemicus* is considered to be an opportunistic pathogen. We suspect that the past medical history of his blunt trauma 2 months earlier was relevant. Infected AA has been attributed to trauma, infection from a contiguous site, blood-borne spread from a preexisting aneurysm due to bacteremia, or embolization in the vasa vasorum due to endocarditis.^4)^ Our review found that 7 patients (cases 3–8, 10) who had underlying medical conditions had a history of undercooked pork or unpasteurized dairy food consumption or direct contact with horses. Moreover, one patient (case 9), who did not have underlying disease, was found to have a history of trauma and worked as a horse trainer. Considering both our case and this review, we suspect that even people working regularly with livestock are unlikely to suffer infection, unless they have underlying medical conditions and/or a triggering event, such as trauma. As previously described, evaluation of patient history and data, including consumption of unpasteurized dairy food, occupation, and extensive contact with animals, is important for an early diagnosis and prompt surgery. Considering the mortality rate of infected AA caused by *S. zooepidemicus*, early diagnosis and prompt surgery are important.

Penicillins, cephalosporins, and gentamicin were commonly used in the cases of our review. In our present case, we administered intravenous cefazolin, followed by long-term suppression with oral amoxicillin/clavulanic acid based on antibiotic susceptibility testing. The duration of antibiotic treatment is still controversial, and we intended to continue oral amoxicillin/clavulanic acid for 6 months, following Yeager et al.^10)^; however, our patient insisted on discontinuing the oral amoxicillin/clavulanic acid prophylaxis after 4 months. Knowing the severity of harm potentially caused by *S. zooepidemicus*, long-term suppression with oral antibiotics for 6 months is probably advisable.^3,10)^ Our review revealed that the patients (cases 6, 10, 11) who received long-term oral antibiotic care had no relapses.

## Conclusion

Here, we report a new case of a previously healthy horse trainer who suffered from an infected AA caused by *S. zooepidemicus*, probably triggered by trauma. We also summarize several cases reported earlier. The reviewed case information suggests that the prognosis of infected AA caused by *S. zooepidemicus* is relatively poor in comparison with infected AA in general. Evaluation of the history of the present illness and patient data, including consumption of unpasteurized dairy food, occupation, and direct contact with animals, is important for an early diagnosis, a prompt surgical treatment, and an appropriate antibiotic therapy.
